# Colorectal Cancer Prediction Based on Weighted Gene Co-Expression Network Analysis and Variational Auto-Encoder

**DOI:** 10.3390/biom10091207

**Published:** 2020-08-20

**Authors:** Dongmei Ai, Yuduo Wang, Xiaoxin Li, Hongfei Pan

**Affiliations:** 1 Basic Experimental Center of Natural Science, University of Science and Technology Beijing, Beijing 100083, China; 2 School of Mathematics and Physics, University of Science and Technology Beijing, Beijing 100083, China; S20190829@xs.ustb.edu.cn (Y.W.); S20180729@xs.ustb.edu.cn (X.L.); S20170825@xs.ustb.edu.cn (H.P.)

**Keywords:** weighted gene co-expression network analysis, variational autoencoder, colorectal cancer, hub genes, classifier

## Abstract

An effective feature extraction method is key to improving the accuracy of a prediction model. From the Gene Expression Omnibus (GEO) database, which includes 13,487 genes, we obtained microarray gene expression data for 238 samples from colorectal cancer (CRC) samples and normal samples. Twelve gene modules were obtained by weighted gene co-expression network analysis (WGCNA) on 173 samples. By calculating the Pearson correlation coefficient (PCC) between the characteristic genes of each module and colorectal cancer, we obtained a key module that was highly correlated with CRC. We screened hub genes from the key module by considering module membership, gene significance, and intramodular connectivity. We selected 10 hub genes as a type of feature for the classifier. We used the variational autoencoder (VAE) for 1159 genes with significantly different expressions and mapped the data into a 10-dimensional representation, as another type of feature for the cancer classifier. The two types of features were applied to the support vector machines (SVM) classifier for CRC. The accuracy was 0.9692 with an AUC of 0.9981. The result shows a high accuracy of the two-step feature extraction method, which includes obtaining hub genes by WGCNA and a 10-dimensional representation by variational autoencoder (VAE).

## 1. Introduction

Colorectal cancer (CRC) is a malignant tumor that ranks third for incidence and second for mortality worldwide [1]. Despite advances in medical technology, most CRC patients go to the hospital because of pain when the cancer is in the middle and late stage. These statistics call for an effective early diagnosis and novel prognostic markers for CRC. Classification models for an accurate screening and diagnosis are required for precision medicine [2]. An effective feature extraction method can avoid the problem of overfitting [3]. The feature extraction method is a key factor which affects the performance of the classifier so as to improve the accuracy of microarray gene expression datasets in predicting CRC.

Researchers have presented many methods for predicting CRC, such as SVM, logistic regression model, discriminative deep belief networks (DDBN), and so on. Zhao et al. selected factors such as the weight, tumor type, and tumor grade as the classification features of a classifier based on logistic regression (LR) and a support vector machine (SVM) [4]. Agesen et al. selected the gene expression data of 13 genes closely related to CRC as classification features to construct a ColoGuideEx classifier, so as to achieve the effective classification of stage II colorectal cancer [5]. Gabere et al. used the gene expression data of 30 genes closely related to CRC as classification features of SVM for predicting CRC, the accuracy of classifier meeting 0.95 [6]. Cubiella et al. applied 11 factors, such as age, gender, faecal haemoglobin, and carcinoembryonic antigen to a logistic regression model for screening CRC [7]. Karabulut et al. constructed discriminative deep belief networks (DDBN) with gene expression profile data for colorectal cancer prediction [8]. Yong et al. applied the miRNA expression profile data of differential genes in cancer tissue and blood samples to a logistic regression model for predicting CRC [9].

To screen and diagnose cancer, researchers usually focus on genes whose expression are significantly different between cancer samples and normal samples. Pathogenic cancer genes are usually correlated, and the occurrence of cancer is the result of an abnormal expression of multiple genes [10]. If the expression of certain genes in a physiological process is similar between different tissues, then these genes are functionally related, and thus they can be treated as a whole group [11]. Gene modules are made by grouping genes according to relevance, and genes with a higher correlation were, accordingly, classified as one type by weighted gene co-expression network analysis (WGCNA). WGCNA is similar to cluster analysis, but WGCNA is a more biologically significant statistical method. WGCNA can be used for finding clusters (modules) of highly correlated genes. It can also be used to find modules that are closely related to disease. WGCNA has been used to find genes related to lung squamous cell carcinoma and colorectal cancer [12,13].

Microarray gene expression data have the characteristics of a high dimension and small sample size, and there are also a large number of redundant genes that will cause a curse of dimensionality, interfere with the diagnosis, and reduce the accuracy of a classifier. Thus, it is important to reduce the dimensions of gene expression data. Common methods to reduce the dimensionality are a principal component analysis [14], Fisher’s linear discriminant analysis [15], and autoencoder [16]. Autoencoder is a type of unsupervised learning technology that uses neural networks to learn to express low-dimension features from high-dimensional features. In many tasks, the autoencoder has a better effect than principal component analysis. There are many improved autoencoder algorithms, such as stack autoencoder [17], sparse autoencoder [18], denoising autoencoder [19], and variational autoencoder (VAE). AE just maps the data to a function. VAE map the data information to a distribution, and the distribution can effectively summarize the information on this kind of data [20]. VAE successfully reduced the dimension of microarray gene expression data, and the effective features closely related to cancer were extracted from microarray gene expression data [21,22].

We used WGCNA to construct co-expressed gene modules and identified a key gene module that was most closely related to cancer. We analyzed the hub genes in this key module. Hub genes may serve as effective early diagnostic and prognostic markers for CRC. We used the hub genes as a type of feature classifier. We applied the VAE to genes with significantly different expression data, and we mapped the results to a 10-dimensional representation as another type of cancer feature classifier.

## 2. Materials and Methods

### 2.1. CRC Microarray Datasets

We downloaded the gene expression datasets GSE8671 [23], GSE9348 [24], GSE23878 [25], and GSE37364 [26] from the GPL570 (Affymetrix Human Genome U133 Plus 2.0 Array) platform, and these included 106 normal samples and 132 colorectal cancer samples (Supplementary Information Table S1).

### 2.2. Datasets Preprocessing

For repeated probe values, we calculated their median as the new value of the probe to ensure the uniqueness of the probe. The uploaders of the four datasets normalized each chip. Two datasets, GSE23878 and GSE37364, were logarithmically processed. We first processed the dataset without logarithm; all data were the result of $log_{2}\left(count\right),$ with the count representing the amount of gene expression [27]. Each dataset in the GEO database has a small sample size, and statistical analysis performed on small samples is usually not robust [28]. Thus, it is necessary to perform a joint analysis of the gene expression data from many different sources. The four expression datasets were combined into one dataset according to the same gene symbols, and the expression data of 13,487 genes from 238 samples were obtained.

However, different conditions can lead to batch effects [29]. A number of approaches can be used to remove batch effects between different datasets, such as the Empirical Bayes method (ComBat) [30], singular value decomposition (SVD) [31], and distance weighted discrimination (DWD) [32]. The empirical Bayesian algorithm can adjust the batch effect in microarray expression data [33].

After combining the four sets of GSE data, we used the sva package in R language based on the empirical Bayesian framework [34] to remove the batch effect for the subsequent statistical analysis.

To remove the batch effect, we assume that the measured expression value of gene i in sample j of batch g can be expressed in a general form as follows:

(1) $$x_{ij}=x_{ij}^{\prime }+b_{ij}^{x}+\varepsilon_{ij}^{x}$$

Then, we standardize it:

(2) $$\hat{x_{ij}}=\frac{x_{ij}-\overset{-}{x_{i}}}{\sigma_{x_{i}}}$$

In the above formula, $\overset{—}{x_{i}}$ is the average value of the expression value from gene i in all samples; $\sigma_{x_{i}}$ is the standard deviation for $x_{i}$ of the expression value from gene i in all samples.

### 2.3. Screening of Differentially Expressed Genes

An important task of gene expression profiling analysis is to perform a variable screening based on known data in order to find genes with significantly different expressions between samples. The typical practice is to use a combination of fold change [35] and T test [36]. The most commonly used tools for differential gene expression analysis are DESeq2 [37], EdgeR [38], and limma [39]. We used the limma package, the P-values were corrected using the FDR correction toolkit in R language. We selected genes with significant differences in choice (adj. *p* < 0.05) and more than double the difference in gene expression between normal and cancer tissues ($\left|\log_{2}FC\right|>1$) to serve as differentially expressed genes.

### 2.4. Construction of Co-Expression Network and Identification of Key Modules

The scale-free gene network condition should be satisfied before conducting WGCNA. A hierarchical clustering tree (module) is built based on the correlation matrix of the gene co-expression, adjacency function, and the dissimilarity measurements of different nodes. The dynamic tree cut method can identify more accurately and more biologically significant co-expression modules [40]. A key module with a high correlation with a specific phenotype or disease can be extracted from these co-expression modules. The hub genes are identified according to the internal connectivity of genes in the key module and the correlation between genes and feature vectors of the key module [41,42].

### 2.5. Mining Hub Genes from the Key Module

The module membership (MM) was calculated via the WGCNA function *signedKME*, which correlated the module eigengene (ME) with gene expression values, thereby quantifying how close a gene was to a given module. The correlation between individual genes and a biological trait (cancer or normal) was defined as the gene significance (GS). The summation of adjacency performed for all genes in a particular network was calculated as the intramodular connectivity (K.in).

Hub genes in a module usually have high GS, high MM, and high K.in [43,44]. Therefore, we first selected genes with GS > 0.5 and MM > 0.8. Then, we screened for genes with significantly different expressions in normal and colorectal cancer samples from the genes that met the above conditions. Finally, we sorted the selected genes into descending order according to the K.in value and considered the top ten genes as the hub genes for this analysis. This approach provided a new method for reducing the dimension issues that often accompany, and potentially interfere with, cancer prediction and biological significance.

### 2.6. Dimension Reduction with VAE

Autoencoder is a type of artificial neural network used to learn the compressed representation for a set of data. This neural network has the same number of neuron nodes in the input layer and output layer, but fewer in the hidden layer. The hidden layer variable of the VAE can learn the distribution of the original data to ensure that the hidden layer can better abstract the characteristics of the input data. Variable inference and reparameterization are used to reduce the dimension of gene expression data. We extracted low-dimensional information from gene expression data with VAE and used this low-dimensional information as a type of feature classifier for screening cancer.

Our pipeline for using WGCNA in order to identify hub genes of colorectal cancer and for using VAE in order to identify a 10-dimensional representation can be freely accessed from GitHub at https://github.com/gutmicrobes/WGCNA-VAE.git.

### 2.7. SVM

Support Vector Machine (SVM) is a class of generalized linear classifier, which has good robustness, with supervised learning that carries out a binary classification of data [45]. The advantages of SVM in predicting are quantitative and qualitative [46]. Researchers have used linear SVM to predict CRC [4]. We analyzed three data sets, GSE8671, GSE9348, and GSE23878, and we selected 10 hub genes and the 10-dimensional representation from the variational autoencoder (VAE) for 1166 genes with significantly different expressions as the classifier for CRC.

## 3. Results and Discussion

### 3.1. Extraction of Differential Genes

**Figure 1:**
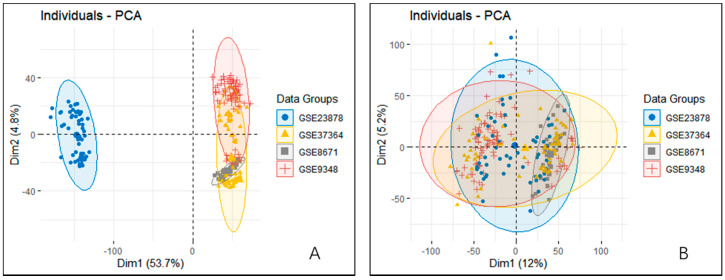
PCA analysis of four datasets before and after the batch effect correction. (**A**) PCA analysis of four datasets before batch effect correction. Dataset GSE23878 was significantly different from the other three datasets; (**B**) PCA analysis of four datasets after batch effect correction. The batch effects of the four datasets were basically eliminated after the correction.

We integrated four datasets as the sample set for the final calculations. To test for the existence of batch effects and whether the four dataset samples were completely separated or whether they had some continuity, we performed a principal components analysis (PCA) on all gene expression data (Figure 1A). The four sample sets were not completely continuous; in particular, dataset GSE23878 was significantly different from the other datasets. We then performed a batch effect correction, and a PCA analysis was performed again on the corrected data. The results in Figure 1B show that the separation among the four datasets after the correction was essentially eliminated.

We divided gene expression data that came from the datasets GSE8671, GSE9348, and GSE23878 into normal and cancer groups for a differential analysis. A volcano plot shown in Figure S1 of the Supplementary Information shows the distribution of genes expressed between cancer and normal tissue.

An important task in gene expression profiling is to perform variable screening based on known data in order to find meaningful differential genes. Accordingly, we performed a differential analysis on the gene expression profiling data after removing the batch effect, as described above. We identified 1159 significantly altered genes from four GEO datasets, of which 419 genes were upregulated and 740 genes were downregulated.

### 3.2. Extracting Hub Gene by Weighted Correlation Network Analysis

#### 3.2.1. Soft Threshold Screening

We used the *pickSoftThreshold* grid search function to calculate the β value of the scale-free network. Table S2 in the Supplementary Information summarizes the parameters corresponding to β. Included are the square of the correlation coefficient between log(p(k)) and log(k) (SET.R.sq), slope and correlation coefficient of the truncation index model (*truncated.R.sq*), average degree of connectivity (*mean.k*), median degree of connectivity (*median.k*), and maximum degree of connectivity (*max.k*). The main parameters we considered in the table were *SET.R.sq* and *mean.k*.

As the soft threshold increased, the *SET.R.sq* and *mean.k* showed corresponding changes (Supplementary Information, Figure S2). Generally, *SET.R.sq* must be greater than 0.85; that is, the network was considered as meeting the requirement of a no scale distribution. When β=12, the network met the requirements of a scale-free distribution (Figure S2). Therefore, we set the threshold β to 12.

#### 3.2.2. Gene Module Construction

**Figure 2:**
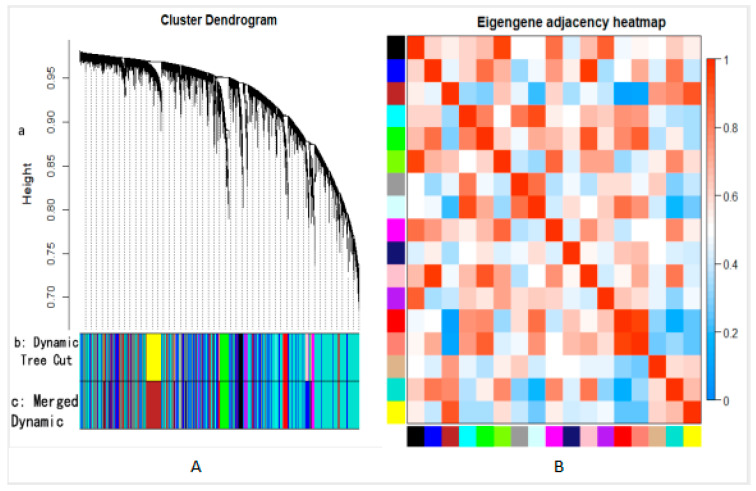
(**A**) System clustering tree of all samples after classification. Part a is the clustering tree constructed by genes, part b is the gene module obtained by clustering, and part c is the gene module obtained by combining similar expression patterns; (**B**) The thermal map of the relationship between the eigenvalues of different modules.

We calculated the topological matrix and the adjacency matrix according to the soft threshold and then transformed the topological matrix into a dissimilarity matrix. We clustered according to the degree of dissimilarity in order to obtain the system clustering tree. In the clustering tree, each “twig” corresponded to a module, and each piece of “leaf” represented a gene in the module. All samples were clustered so as to form 17 modules, the smallest of which contained 36 genes (Figure 2A). At most, the modules contained 6377 genes, with an average of 814 genes per module.

Each co-expressed gene module was a set of genes with similar expression patterns, and all gene expression amounts in the module were then combined in order to obtain a “characteristic value,” which we called a module characteristic value. The module eigenvalue was the result of the operation of the common eigenvalues of all genes in the module. We used the module eigenvalue to calculate the correlation, and the thermal map is plotted in Figure 2B.

Figure 2B shows a certain correlation between different modules. Modules with a high correlation can be further merged into the same module. Accordingly, we used the dynamic pruning method to further merge modules with a significant correlation into the same module. To form 12 modules, we defined a minimum of 30 genes per module and combined modules with a correlation coefficient greater than 0.8. Table 1 shows the 12 modules and the number of genes included.

To analyze the relationship between gene modules and cancer, we quantified cancer patients as 1, normal people as 0, and we calculated the ME for each module; all genes in the module are represented by ME. We calculated the Pearson correlation coefficient (PCC) between ME and cancer (Table 2) [47]. With the exception of the midnight blue module, all other modules were significantly correlated with cancer. The results of quantitatively analyzing the correlation between cancer and the module eigengene indicated that the gene module division after pruning had a biological significance.

In Table 2, a large PCC and a small *p*-value indicated a stronger relationship between the module and cancer. The PCC between the MEturquoise module and cancer was the highest, and the *p*-value was the lowest, making the correlation biologically significant. These genes were the most important in the module that distinguished cancer from normal tissue.

#### 3.2.3. Hub Gene Identification

According to the coefficient between ME and cancer, MEturquoise became our module of interest, and it contained 6865 genes. Therefore, we further mined the hub genes from this module.

A hub gene in a module usually has a high GS, high MM, and high K.in. Therefore, we first selected genes with GS > 0.5 and MM > 0.8. These conditions were met by 191 genes, of which 165 had significant differences between cancer and normal samples.

Then, to explore the functional association between gene modules and colorectal cancer, we performed a Gene Ontology and KEGG pathway enrichment analysis on the aforesaid 165 genes (see Supplementary Information, Figure S3 and Figure S4).

Finally, we selected the top 10 genes with the largest K.in as the hub genes for this study (Table 3).

*GUCA2B* (Guanylate Cyclase Activator 2B), a protein coding gene, is a physiological regulator of intestinal fluid and electrolyte transport. Nagaraj et al. considered *GUCA2B* as a noninvasive biomarker for the early detection of colorectal cancer [48]. Gene Ontology annotations for this gene include calcium-sensitive guanylate cyclase activator activity and guanylate cyclase activator activity. The *GUCA2A* gene is an important paralog of *GUCA2B*.

*CDK4* (cyclin-dependent kinase 4) is important for cell cycle G1 phase progression. It is a regulator of the cell cycle. Lee et al. use *CDK4/6* inhibitors to inhibit the proliferation of colorectal cancer cells effectively [49].

*TRIP13* (thyroid hormone receptor interactor 13) messenger RNA was highly expressed in multiple CRC tissues. The depletion of *TRIP13* in CRC cells suppressed cell proliferation, migration, and invasion [50].

As for *EIF3B* (eukaryotic translation initiation factor 3 subunit B), the researchers found that the silencing of *EIF3B* gene expression could significantly inhibit the colorectal cancer cell proliferation rate and clonability [51].

The *CDH3* gene encodes cadherin, a type of cell adhesion molecule that is important in the formation of adherens junctions that bind cells [52]. *CDH3* is located in a gene cluster of chromosome 16 that is involved in breast cancer and prostate cancer. An abnormal expression of *CDH3* protein is also observed in cervical cancer. This gene is also associated with adolescent macular dystrophy and poor ectodermal release. *CDH3* is overexpressed in colorectal tumors, and it has potential as a serum marker for colorectal cancer surveillance [53].

*GUCA2A* (Guanylate Cyclase Activator 2A) is a protein-coding gene. Among its related pathways are the Metabolism and Myometrial Relaxation and Contraction Pathways. Gene Ontology annotations for this gene include hormonal activity and guanylate cyclase activator activity. Zhang et al. revealed that *GUCA2A* was downregulated in CRC tissues [54].

### 3.3. Realization of VAE

We designed a VAE model to extract low-dimensional information from gene expression data as a type of feature for the analysis and prediction of cancer. There were more nodes between the input layer and the first hidden layer, with 680,000 parameters needing training. If we had extracted features directly from more than 10,000 genes with expression data, we would have needed to reduce the number of hidden layers of the model and the number of neuron nodes in the hidden layer, thereby reducing accuracy. Therefore, the input data were genes with significant differences. We adopted a neural network model with an input-output layer and five hidden layers. In the VAE, the numbers of hidden layer nodes in the model were 584, 100, 10, 100, and 584, and the input layer and output layer nodes were 1159. The learning rate was 0.0005, batch size was 20, and epoch was 6. To measure the dimensionality reduction effect of the model, the last 65 samples of the gene expression data were used as the test set, and all the other samples were used as the training set. We determined that the appropriate epoch to ensure the stable convergence of the model was six by calculating the reconstruction error and the loss function of the model on the test set. Finally, we used a VAE model to successfully scale the gene expression data into 10 dimensions and use them as one of the characteristics of the classifier.

### 3.4. Analysis Results from the Model of Cancer Prediction

We used the classifier SVM to identify a classification method suitable for the dataset described here. We used three datasets as the training set: GSE8671, GSE9348, and GSE23878; meanwhile, the dataset GSE37364 was the test set. The training set obtained 68 healthy samples and 105 cancer samples, and the test set obtained 38 healthy samples and 27 cancer samples. The training set and the test set basically balanced the positive and negative samples. SVMs are more flexible in binary tasks and have a variety of kernel functions to choose from. A new taxonomic data set was constructed by combining the hub genes with the expression of the low-dimensional features of the VAE. SVM was used to classify the new classification dataset, with a test accuracy of 0.9692 and AUC of 0.9981. The SVM classified the 65 samples in the test set exactly. The two-step dimensionality reduction method greatly improved the classification accuracy.

## 4. Conclusions

We obtained 1166 genes with significant differences in gene expression from 238 normal and cancer samples. The number of genes obtained by differential analysis was relatively large, and many of these genes interact. We used WGCNA to construct a scale-free network of gene co-expression data. WGCNA divided genes into different modules and found that the genes in each module were similar in expression. On the basis of a correlation analysis between the module eigengene and the samples, we identified the MEturquoise module, which may have an important function in carcinogenesis. Our results showed that the genes in this module were different from those in the other modules. That is, *GUCA2A*, *GUCA2B*, *CDH3*, and other hub genes were highly correlated with the occurrence of CRC. We used 10 hub genes as one type of feature for CRC prediction. We also used VAE to reduce the gene spectrum data to 10 dimensions and derive another type of feature for the CRC prediction model. The accuracy of the CRC prediction model was 0.9692.

## Figures and Tables

**Table 1:** Gene module and number of corresponding genes.

Color	Tan	Brown	Turquoise	Blue	Green	Purple
Number	85	2799	6377	2636	400	153
**Color**	**Black**	**Magenta**	**Midnight Blue**	**Red**	**Cyan**	**Grey60**
Number	330	175	60	318	118	36

**Table 2:** The PCC between each module and cancer after pruning.

	MEtan	MEbrown	MEturquoise	MEblue	MEgreen	MEpurple
**PCC**	−0.1285	−0.3052	−0.9251	−0.7075	−0.2017	0.3457
***p*-value**	0.0920	0.0000	0.0000	0.0000	0.0078	0.0000
	**MEblack**	**MEmagenta**	**MEblue**	**MEred**	**MEcyan**	**MEgrey60**
**PCC**	−0.4263	−0.5753	0.0609	0.5127	0.2944	0.3082
***p*-value**	0.0000	0.0000	0.4260	0.0000	0.0000	0.0000

**Table 3:** Top 10 genes with the highest intramodular connectivity.

GENE NAME	logFC	adj.P.Val	GS	MM.Turquoise	K.in
**CDK4**	1.3042	2.40 × 10^−39^	0.8128	−0.9076	933.8071
**CDH3**	6.3918	2.92 × 10^−80^	0.9417	−0.9206	922.1908
**DKC1**	1.3517	3.34 × 10^−41^	0.8226	−0.9055	918.2304
**UBE2S**	1.9085	7.08 × 10^−40^	0.8118	−0.8997	906.0183
**GUCA2B**	−6.4444	6.12 × 10^−53^	0.8723	0.9075	895.3242
**UBE2C**	2.1435	9.16 × 10^−52^	0.8706	−0.9122	895.1551
**EIF3B**	1.3173	1.02 × 10^−43^	0.8368	−0.8970	894.6524
**TRIP13**	1.9410	2.07 × 10^−46^	0.8474	−0.8905	890.4061
**GUCA2A**	−5.3201	2.29 × 10^−50^	0.8628	0.8628	887.2683
**GTF3A**	1.4126	5.21 × 10^−39^	0.8085	−0.8173	883.3120

## Supplementary Materials

The following are available online at https://www.mdpi.com/2218-273X/10/9/1207/s1, Figure S1: Volcano map of differential genes between colorectal cancer sample and normal sample. Figure S2: Soft threshold Screening. Figure S3: GO Functional enrichment analysis. Figure S4: KEGG function enrichment analysis. Table S1: Sample distribution of four datasets. Table S2: Soft Threshold Screening for Network Construction.
